# First detection of *Colletotrichumfructicola* (Ascomycota) on horsehair worms (Nematomorpha)

**DOI:** 10.3897/BDJ.9.e72798

**Published:** 2021-09-23

**Authors:** Mattia De Vivo, Wen-Hong Wang, Ko-Hsuan Chen, Jen-Pan Huang

**Affiliations:** 1 Biodiversity Research Center, Academia Sinica, Taipei, Taiwan Biodiversity Research Center, Academia Sinica Taipei Taiwan; 2 Department of Life Science, National Taiwan Normal University, Taipei, Taiwan Department of Life Science, National Taiwan Normal University Taipei Taiwan; 3 Biodiversity Program, Taiwan International Graduate Program, Academia Sinica and National Taiwan Normal University, Taipei, Taiwan Biodiversity Program, Taiwan International Graduate Program, Academia Sinica and National Taiwan Normal University Taipei Taiwan

**Keywords:** Nematomorpha, Taiwan, *
Colletotrichum
*, horsehair worms, *
Chordodes formosanus
*, fungi

## Abstract

Fungal members of *Colletotrichum* (Ascomycota) were found to be associated with *Chordodesformosanus*, one of the three currently known horsehair worm (Nematomorpha) species in Taiwan. The fungi were identified as *Colletotrichumfructicola*, which is mostly known as a plant pathogen, through the use of the nuclear ribosomal internal transcribed spacer and partial large subunit (nrITS + nrLSU) and glyceraldehyde-3-phosphate dehydrogenase (GAPDH) DNA sequences. To our knowledge, this report represents both the first records for *Colletotrichum* associated with hairworms and for fungi on Nematomorpha. These findings expand the knowledge on the ecological relationships of both clades.

## Introduction

The phylum Nematomorpha (which includes animals commonly known as “horsehair worms” or “hairworms”) is regarded as one of the most understudied animal groups, both from taxonomic and ecological perspectives (Schmidt-Rhaesa 2012). Most species have a complex life cycle that involves a larva encysting inside a freshwater intermediate host (i.e. usually an insect larva), a juvenile phase in which they parasitise terrestrial arthropods and a free-living adult freshwater stage (Schmidt-Rhaesa 2012, Bolek et al. 2015). However, some species have only freshwater hosts and a free-living freshwater adult stage (Schmidt-Rhaesa 2012). In addition to the freshwater ones, there are marine horsehair worms (all belonging to the genus *Nectonema*) that parasitise crustaceans as juveniles and live in surface seawater as adults (Schmidt-Rhaesa 2012, Kakui et al. 2021). Moreover, two recently-described Nematomorpha live in terrestrial wet environments in the adult phase (Anaya et al. 2019, Chiu et al. 2020).

Although we have some knowledge on Nematomorpha’s life history, there are very few studies on commensals, symbionts and parasites of hairworms. In addition, there are no reports of potential horsehair worm pathogens, both prokaryotic and eukaryotic, in literature (Schmidt-Rhaesa 2012, Bolek et al. 2015). The lack of data stems from two factors that make hairworms hard to observe: their generally reclusive behaviour (freshwater species tend to hide under rocks, fallen leaves and branches) and the absence of standardised protocols for sampling them. Moreover, few researchers study Nematomorpha due to their low medical and economical importance (Schmidt-Rhaesa 2012, Bolek et al. 2015).

Here we provide morphological and molecular evidence for the presence of fungi resembling *Colletotrichum* species (Ascomycota) living on and inside the body of *Chordodesformosanus*, one of the three described Taiwanese hairworm species (Chiu et al. 2020). The genus *Colletotrichum* mostly includes plant-associated (i.e. pathogen or endophytes) taxa with a broad host range. Some species also parasitise commercially-valuable crops (Cannon et al. 2012, Weir et al. 2012). However, species infecting sea turtles (Manire et al. 2002), cats (Winter et al. 2010), scale insects (Marcelino et al. 2008, Wynns et al. 2020) and humans (Cano et al. 2004, Lin et al. 2015) were described occasionally.

## Materials and Methods

**Figure 1.**
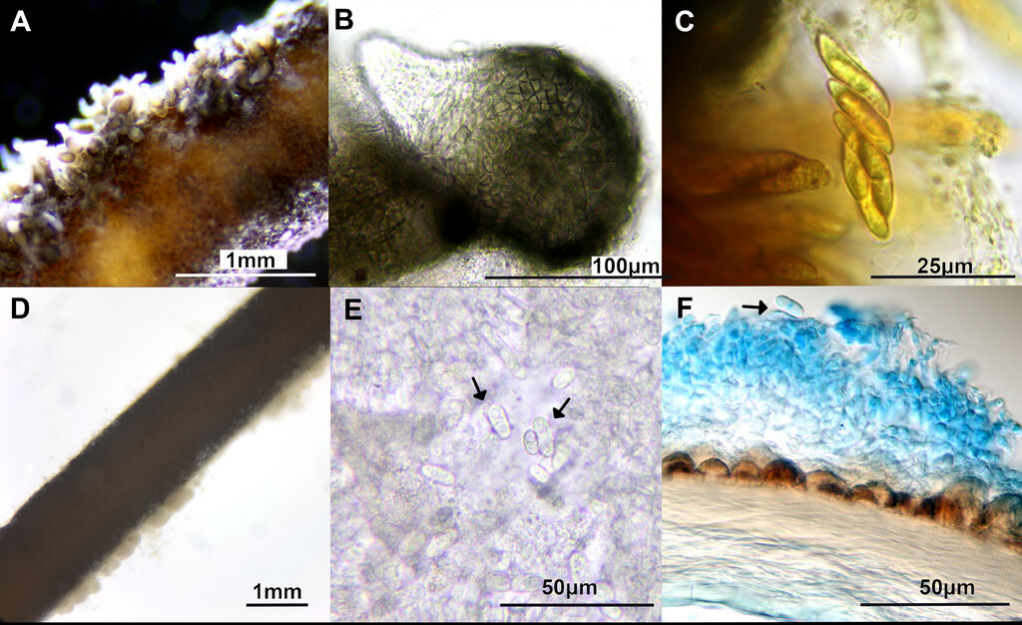
Microscopic investigation of *Chordodesformosanus* infected with *Colletotrichumfructicola.* **A** Multiple perithecia of *C.fructicola* on the cuticle of hairworm; **B** Perithecium on the cuticle; **C** Ascospores in an ascus, dyed with Lugol’s Solution; **D** White acervuli on the hairworm cuticle; **E** Conidia and sporulating structures; **F** Conidia (shown with arrows) and sporulating structures on the cuticle. A-C correspond to E6 (sexual state), which is also respresented in Fig. 2 and D-F correspond to E5 (asexual state).

Four free-living adults of *C.formosanus* were collected in Wufengqi Waterfall area in Yilan County, Taiwan (24°49'59.6"N, 121°44'47.3"E) on 11 August 2020 (Suppl. material 1). After 10 days in a tank with a mixture of tap water and water collected from the collection site, fungi visibly started to develop on the hairworms. After one month and ten days, with the water replaced with tap water only, the fungi were widespread all over the worms’ cuticles (Fig. 1A and D). Despite this, three worms were alive at the time of fungal investigation.

**Figure 2.**
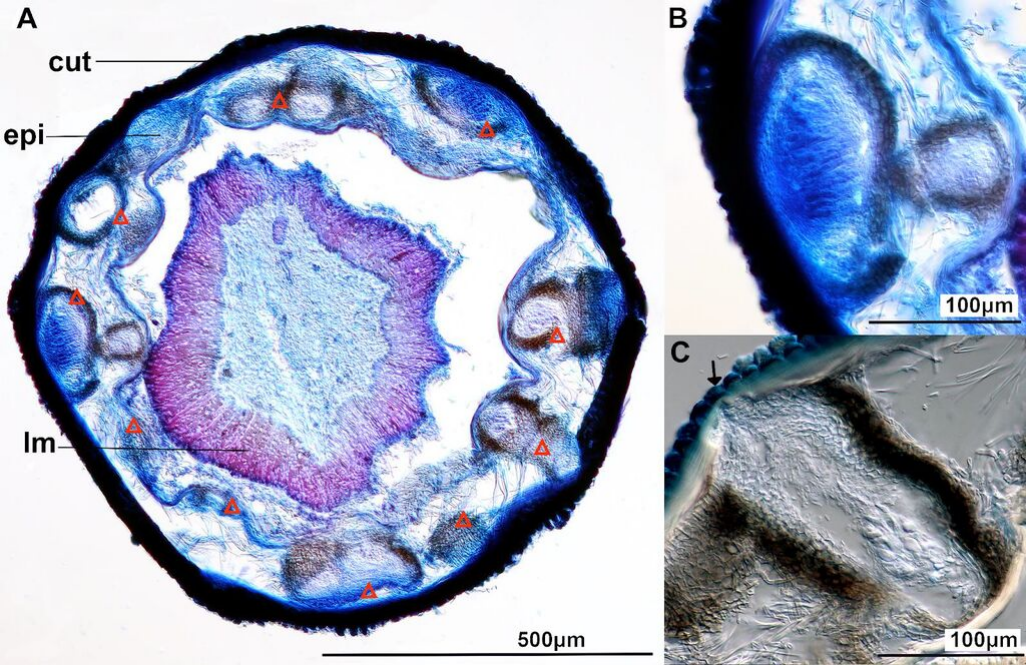
Dissection of *Chordodesformosanus* infected with *Colletotrichumfructicola* (sexual state) **A** Cross section of the hairworm showing perithecia lined up underneath the cuticle. Stained with Trypan blue. Abbreviations of hairworm structures: cuticle (cut), epidermis (epi) and longitudinal muscles (lm). The muscles detached from the epidermis due to dehydration of the tissues. Arrow = perithecia. Further cross-sections are present in Suppl. material 2; **B** Cross section of a perithecium with asci and ascospores inside (enlarged fromFig. 2A); **C** Cross section of the hairworm showing a perithecium protruding the cuticle layer.

The worms were investigated and two fungal structures (i.e. acervulus and perithecium) were dissected for further microscopic and molecular assessment (Fig. 1, Fig. 2 and Suppl. material 2). The investigation was performed with a dissecting microscope (Leica S9D) and a compound microscope (Nikon Eclipse N1). In addition, the regions of the worm with obvious fungal infection were sectioned with a tissue dissector (Leica CM3050 S Cryostat). Fungal perithecia were on and beneath the worm cuticle (Fig. 2). Asexual sporulating structures bearing conidia were found on the surface of the cuticle (Fig. 1D-F). Two of the hairworm specimens were deposited at the Biodiversity Research Museum, Academia Sinica, Taipei (collection IDs: ASIZ01000033 and ASIZ01000034).

The aforementioned fungal structures were selected for DNA extraction and amplified with several universal primer sets for amplyfing four genes: ITS1F 5’ CTTGGTCATTTAGAGGAAGTAA 3’ and LR3 5’ CCGTGTTTCAAGACGGG 3’ or ITS4 5’ TCCTCCGCTTATTGATATGC 3’ (White et al. 1990, Vilgalys and Hester 1990), which targeted nuclear ribosomal internal transcribed spacer and partial large subunit (nrITS + nrLSU); GDF3 5’ GCCGTCAACGACCCCTTCATTGA 3’ and GDR3 5’ TTCTCGTTGACACCCATCACGTACATG 3’ (Chung et al. 2020) for targeting glyceraldehyde 3-phosphate dehydrogenase (GAPDH); CHS-79F 5’ TGGGGCAAGGATGCTTGGAAGAAG 3’ and CHS-345R 5’ TGGAAGAACCATCTGTGAGAGTTG 3’ (Carbone and Kohn 1999) for chitin synthase (CHS-1); CL1C 5’ GAATTCAAGGAGGCCTTCTC 3’ and CL2C 5’ CTTCTGCATCATGAGCTGGAC 3’ (Weir et al. 2012) for calmodulin (CAL). For DNA extraction, we placed tissues in Tris-EDTA (TE) buffer (50 µl) and stored them at -20°C. Then, the frozen tubes were placed into an ultrasonic bath for 30 sec and in a thermal cycler at 95°C for 10 min to break the cell wall.

PCR was undertaken by using Illustra™puReTaq Ready-To-Go PCR Beads (GE Healthcare, United Kingdom) with 1 µl of forward and reverse primers (for a total of 2 µl), 2 µl of DNA and 21 µl of ddH_2_O. The thermal cycler was set with an initial cycle at 94°C for 5 min, then 35 cycles with 94°C for 30 s, 52°C (ITS)/58 °C (other genes) for 1 min and 72°C for 90 sec. Extension was done at 72°C for 10 min. The amplicons were sequenced by both the forward and reverse primers.

The sequences derived from both directions were manually trimmed of the poor-quality reads with MEGA X 10.1.8 (Kumar et al. 2018) and a consensus sequence was saved. The sequences were submitted to NCBI GenBank with the following accession numbers: E5 = MW714777, E6 = MW714778 for the ITS sequences; E5=MZ965243, E6=MZ965244 for the GAPDH ones. CHS-1 and CAL were successfully amplified only for E6 and their sequences have the following accession numbers: MZ965245 for CHS-1, MZ965246 for CAL.

We then conducted a BLASTn search (Altschul et al. 1990) with default settings for finding similar sequences in GenBank. After identifying a genus with high degree of sequence similarity through BLASTn, we retrieved sequences of species inside that clade from GenBank (Suppl. material 3) and we reconstructed a phylogenetic tree. Specifically, sequences alignment was performed using the L-INS-i algorithm in MAFFT 7.471 (Katoh and Standley 2013) and Maximum Likelihood phylogenies for concatened genes were subsequently reconstructed using ModelTest and RAxML-NG implemented in raxmlGUI (Edler et al. 2021). Gene concatenation was undertaken by using SequenceMatrix (Vaidya et al. 2011). The trees were visualised with FigTree 1.4.4 (Rambaut 2018).

## Results and Discussion

**Figure 3.**
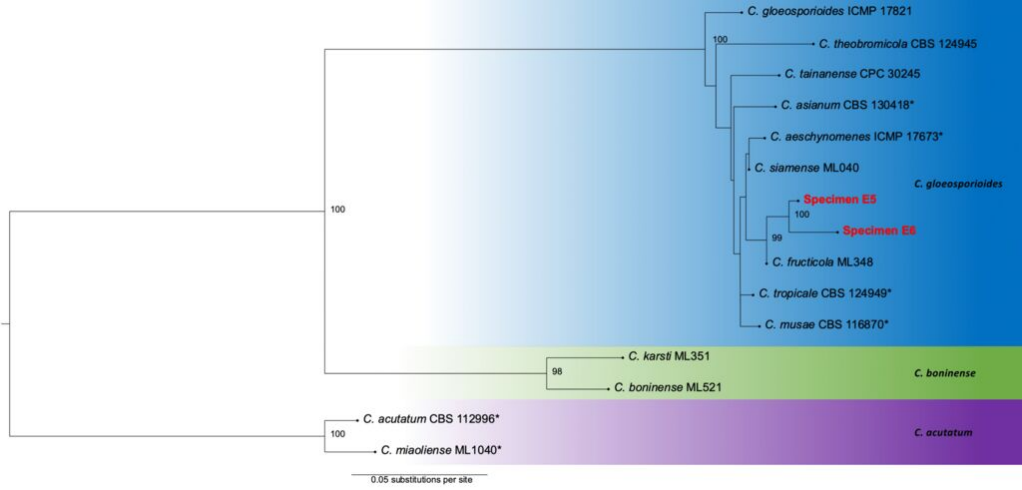
Maximum Likelihood phylogenetic tree built by using concatenated ITS and GAPDH sequences. Specimens E5 and E6 were collected for this study and are emphasised in bold and red font. Bootstrap values ≥ 70 are shown. The names of species complexes are shown on the right. Strain number for sequences taken from GenBank are shown. Strains with the * mark are the ex-type strains. Accession numbers for the gene sequences used are available in Suppl. material 3. A picture of the species tree made with concatenated ITS, GAPDH, CHS-1 and CAL genes with specimen E6 is present in Suppl. material 4.

The fungus E6 (Fig. 3) had obpyriform perithecia, with colours ranging from black to brown, paler towards the ostiolar neck, without hairs (Fig. 1A-C). Asci were unitunicate with nonamyloid apex, with hyaline and unicellular ascospores, around 15 μm long (Fig. 1C). These features represent the sexual reproductive structures of the fungal genus *Glomerella* which is regarded as the sexual state of genus *Colletotrichum* (Cannon et al. 2012). The other fungus E5 appeared to be at its asexual stage and produced white acervuli bearing hyaline conidia (Fig. 1D-F). The phylogenetic results of the concateneted ITS and GAPDH tree showed that these fungi were *Colletotrichumfructicola* individuals (Fig. 3; Suppl. material 4), which is a taxon belonging to the *Colletotrichumgloeosporioides* species complex (Weir et al. 2012).

In Taiwan, *Colletotrichum* species are mostly known for causing anthracnose in different kind of plantations (Sun et al. 2019, Damm et al. 2020, Wu et al. 2020, Chung et al. 2020). There is also a reported cutaneous infection on a human caused by *C.gloeosporioides* (Lin et al. 2015). From what concerns *C.fructicola*, it has been recognised as a pathogen of strawberry, mango and tea in the Island (Wu et al. 2020, Chung et al. 2020, Lin et al. 2021), but it has also been reported on other crops worldwide (Weir et al. 2012).

Fungi in the phylum Ascomycota are known to be resilient and they can pass from soil to aquatic environments (Jessup et al. 2004, Rypien et al. 2008, Sarmiento-Ramírez et al. 2010, Fisher et al. 2012); this trait is also present in *Colletotrichum* species, which have been reported both from seawater and freshwater organisms (Smith et al. 1989, Manire et al. 2002). In addition to this, all the horsehair worms are known not to feed in the adult stage (Schmidt-Rhaesa 2012). Non-feeding may make the hairworm hosts weaker as time goes by and allow the opportunistic fungi to grow on their cuticle. Further studies will be needed to determine the prevalence of *Colletotrichum* in the wild, apparently healthy Nematomorpha populations and if hairworms can contribute to the spread of the ascomycetous fungi to other organisms, as happens with other related fungal clades (Fisher et al. 2012). Given the possible arising of new diseases from opportunistic pathogens due to environmental change (Nnadi and Carter 2021) and to further understand the chronological order of infection and the pathogenicity of *Colletotrichum* on hairworms, inoculation experiments (for proving Koch’s postulate) combined with histological examination will be required. Besides these considerations, to our knowledge, this is the first report of fungi on horsehair worms. In addition, our report increases the already broad host range of the genus *Colletotrichum*.

## Supplementary Material

91255BB7-D642-57D5-8531-4342C45E1C80 10.3897/BDJ.9.e72798.suppl1 Supplementary material 1 Free living specimens Data type Image Brief description Free living specimens of *Chordodesformosanus*, before the fungi started to be visible File: oo_583596.jpg https://binary.pensoft.net/file/583596 Mattia De Vivo

7E935DBE-389A-5647-80DA-12CDE612BFB8 10.3897/BDJ.9.e72798.suppl2 Supplementary material 2 Further cross sections Data type Multimedia (PDF) Brief description Additional cross sections of the worms File: oo_584098.pdf https://binary.pensoft.net/file/584098 Wen-Hong Wang and Ko-Hsuan Chen

7E70785B-50A8-5BA4-AE77-7EE45C190A39 10.3897/BDJ.9.e72798.suppl3 Supplementary material 3 Accession list of the sequences used in this study Data type GenBank accession numbers (csv file) Brief description Accession numbers of the sequences used for this study File: oo_583511.csv https://binary.pensoft.net/file/583511 Mattia De Vivo

EEEB1A9B-5AE0-5C0D-AE26-57C80524EBDE 10.3897/BDJ.9.e72798.suppl4 Supplementary material 4 4 genes tree Data type Tree (image) Brief description Picture of the concatenated phylogenetic tree based on all the sequenced genes (ITS, GAPDH, CHS-1 and CAL). Bootstrap values ≥ 70 are shown. File: oo_583516.svg https://binary.pensoft.net/file/583516 Mattia De Vivo
