# Supplementary figures and images for: First detection of Colletotrichumfructicola (Ascomycota) on horsehair worms (Nematomorpha)
Source: Biodivers Data J. 2021 Sep 23;9:e72798. doi: 10.3897/BDJ.9.e72798 (PMC8484196; doi:10.3897/BDJ.9.e72798)

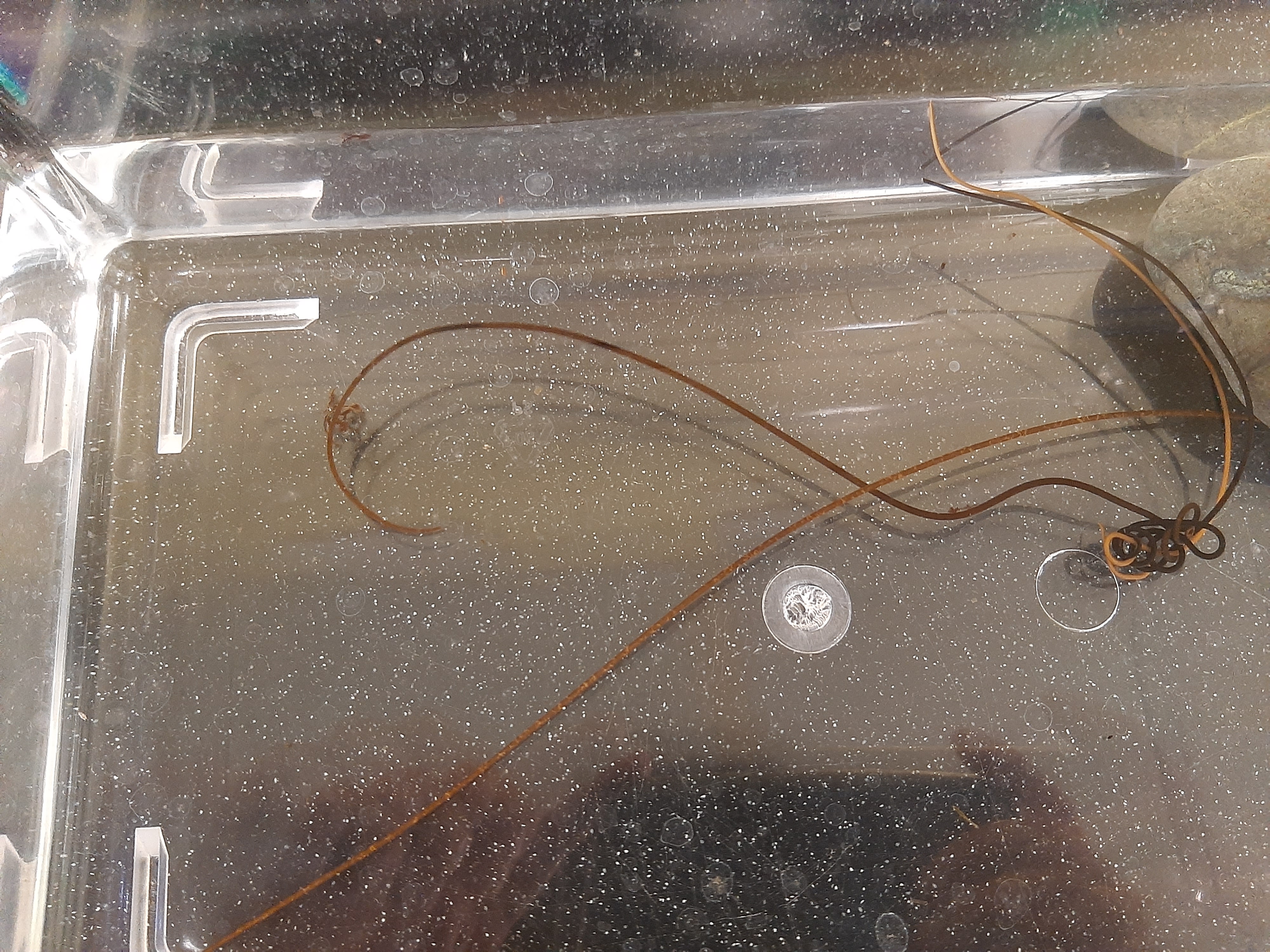

Supplement: Supplementary material 1 — Free living specimens [file bdj-09-e72798-s001.jpg]
