# Supplementary material for: First detection of Colletotrichumfructicola (Ascomycota) on horsehair worms (Nematomorpha)
Source: Biodivers Data J. 2021 Sep 23;9:e72798. doi: 10.3897/BDJ.9.e72798 (PMC8484196; doi:10.3897/BDJ.9.e72798)
Supplement: Supplementary material 2 — Further cross sections [file bdj-09-e72798-s002.pdf]

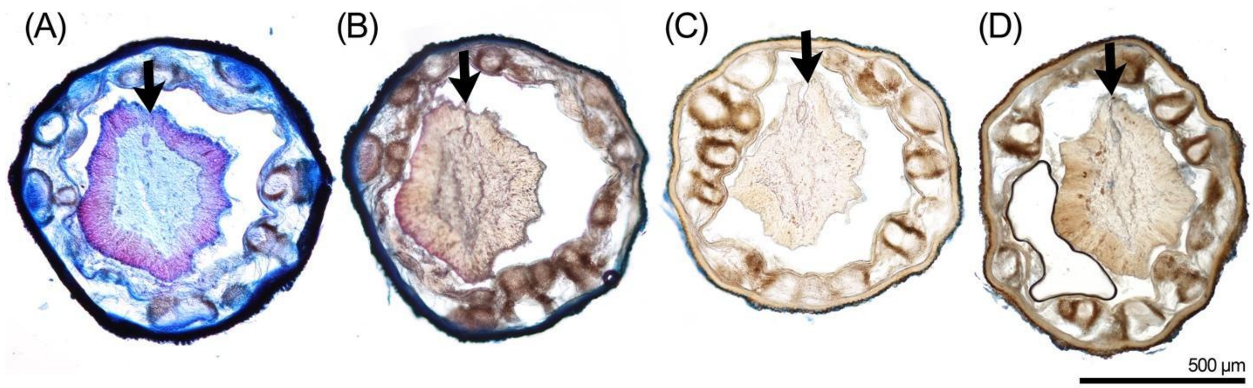

Cross sections of *Chordodes formosanus.* (A) corresponds to Fig. 2A in the main text. Black arrow = Central nerve cord.
